# Enhancement and desert

**DOI:** 10.1177/1470594X18810439

**Published:** 2018-11-14

**Authors:** Thomas Douglas

**Affiliations:** University of Oxford, UK

**Keywords:** achievement, desert, effort, praiseworthiness, moral worth, enhancement, fairness

## Abstract

It is sometimes claimed that those who succeed with the aid of enhancement technologies deserve the rewards associated with their success less, other things being equal, than those who succeed without the aid of such technologies. This claim captures some widely held intuitions, has been implicitly endorsed by participants in social–psychological research and helps to undergird some otherwise puzzling philosophical objections to the use of enhancement technologies. I consider whether it can be provided with a rational basis. I examine three arguments that might be offered in its favour and argue that each either shows only that enhancements undermine desert in special circumstances or succeeds only under assumptions that deprive the appeal to desert of much of its dialectic interest.

Suppose that two budding scientists, Acheson and Baird, gain entry into a highly selective doctoral programme by attaining the two highest scores in the entry examination. Acheson managed this feat only because she used a cognition-enhancing drug – a drug that allowed her to concentrate more intensely than all other candidates. Baird succeeded without such pharmaceutical assistance. Her success was chiefly attributable to her exceptionally conscientious and efficient preparation.

Now suppose that two cyclists, Campbell and Donoghue, tie for first in the Olympic road race. As a result, each receives many accolades and attractive sponsorship deals. Campbell’s exceptional performance was due in part to his use of a new doping substance; Donoghue’s was achieved without doping.

Many would find the pharmaceutically assisted achievements of Acheson and Campbell to be problematic in a way that those of Baird and Donoghue are not. Some have been prepared to generalize from such cases to other enhancements – that is, other biomedical interventions intended to augment human capacities from an already healthy, disease-free level. They have argued that enhancements are *generally* (*viz.* always or almost always)^1^ morally or prudentially undesirable in some respect that more traditional means of augmenting our capacities – such as education and training – are not (Fukuyama, 2002: 172; Sandel, 2007: 92–95).

In what follows I introduce and scrutinize a view that I believe underpins much of this opposition to enhancements. This is the view that (*The Desert Thesis*) Individuals who succeed with the aid of enhancements generally (*viz.*, always or almost always) deserve the goods associated with that success less than otherwise similar individuals who attain the same success without enhancements.

The term ‘success’ is to be understood here as referring to achievement. Two individuals attain the same success if they achieve the same things. ‘The goods associated with that success’ is to be understood as referring both to the achievement itself, if achievements are in themselves good for the achiever, and to any other prudential goods that the achievement confers on the achiever.

The desert thesis has often remained implicit in philosophical discussions of enhancement, and an initial aim of this article is to bring it to the surface; in the first section, I clarify the thesis, and in the second section, I explain why it is worthy of philosophical attention. My primary aim, however, is to *challenge* the thesis. I pursue this aim by examining what I take to be the three most promising arguments that might be offered in support of the thesis: the argument from immoral means (third section), the argument from diminished effort (fourth section) and the argument from shared responsibility (fifth section). I argue that each argument either shows only that enhancements undermine desert *in special circumstances*, thus falling well short of establishing the desert thesis, or succeeds only under assumptions that rob appeals to desert of much of their interest as potential grounds for objections to enhancement. Finally, in the sixth section, I conclude by drawing out some implications of my argument.

## Clarifying the desert thesis

The desert thesis requires three clarifications. These concern (i) the concept of desert that it invokes, (ii) the assumptions about the genesis of achievements on which it is grounded, and (iii) its scope.

First, the concept of desert. The desert thesis makes a claim about the relative desert of two groups of people – those who succeed with the aid of enhancements, and otherwise similar individuals who succeed without enhancements. I have said what this desert is desert *for* – it is desert for the goods associated with the success. But what *kind* of desert is it?

In one commonly deployed sense of desert, to deserve a good is to be entitled to it. If I purchase a non-counterfeit lottery ticket bearing the number 14,376, and this number is subsequently drawn in the lottery, then I am entitled to the prize – I deserve it, in the entitlement sense of desert. But there is another sense of desert in which I may not deserve it: I may not be *worthy* of it; I may be a thoroughly bad person who has already enjoyed far more good luck than befits my flawed nature.

The desert thesis, as I understand it, makes a claim about desert-as-worthiness, not desert-as-entitlement. It holds that those who succeed with the aid of enhancements are generally less worthy of the goods associated with that success than similar individuals who attain the same success without enhancements.

The distinction between desert-as-entitlement and desert-as-worthiness is sometimes equated with a distinction between institutional and pre-institutional desert (e.g., Feinberg, 1970: 55–87). The thought is that entitlements depend on prevailing legal and other institutional arrangements, whereas worthiness does not. However, worthiness can also depend on institutional context. Consider the case of a sporting competition. We can distinguish between the athlete who is entitled to a prize under the rules and conventions governing how the prize should be allocated, and the athlete who is most worthy of this prize. But it is not the case that the latter athlete could be identified without regard for the rules and conventions of the sporting competition. For instance, one factor that is clearly relevant to determining who is worthy of the prize is what the conventionally determined purpose of the competition was. If the purpose of the competition was to test innate running ability, then we may be inclined to say that possessing a high degree of innate running ability is at least one of the factors that could make one worthy of the prize. By contrast, if the point of the competition was to test effort, then innate running ability may be irrelevant to worthiness (Cummiskey, 1987: 18).^2^

Thus, though my focus in what follows will be on worthiness, not entitlement, it does not follow that I will be interested only in a pre-institutional kind of desert; indeed, my argument will at several points rely on the dependence of desert on institutional context.

My second clarification concerns the comparison that the desert thesis draws between those who succeed with the aid of enhancements and the otherwise similar individuals who succeed without enhancements. The thesis holds that the ‘enhanced achievers’ generally deserve the goods associated with their achievements less than the comparator ‘unenhanced achievers.’ As I will sometimes say, omitting reference to the relevant goods and dropping the ‘generally’ qualifier, the thesis holds that enhancement ‘undermines desert’ and that unenhanced achievers are ‘more deserving’ than enhanced achievers. However, the thesis tells us nothing about how, despite the similarities between them, the unenhanced achievers manage to realize the same achievements as the enhanced achievers. Do they augment their abilities through means other than enhancement? Do they simply try harder? Or did they start from a higher level of ability than the unenhanced achievers? We will need to assume *some* difference between the enhanced and the unenhanced achievers that compensates for the deployment of enhancements by the latter, and the nature of this difference could make an important difference to the plausibility of the thesis, since it could affect the degree to which unenhanced achievers deserve the goods in question. It is often thought, for example, that those who succeed largely on the basis of innate ability deserve the rewards they thereby receive less than those who realize the same success despite possessing less innate ability. In order to present the desert thesis in its most plausible light, I will assume that the unenhanced achievers started from the same level of ability as the enhanced achievers and managed to realize the same achievements through either exerting greater effort or augmenting their abilities through the more traditional means of education or training.

Finally, my third clarification concerns the scope of the desert thesis. I limit the scope of the thesis to achievements whose realization without the aid of enhancement contributes positively to the achiever’s desert. That is to say, I limit the thesis to achievements whose realization makes the achiever more deserving of the associated goods than she would have been had she not realized the achievement at all. Not all achievements are like this. Successfully coordinating a terrorist attack does not make one more deserving of any goods associated with doing so; it makes one less deserving of these goods (and, perhaps, more deserving of certain ills). But in many cases, realizing an achievement does make one more deserving of the goods associated with that achievement. This is most clearly so when the achievement is a morally valuable achievement, but it is plausibly also true of some morally neutral achievements. For example, many would hold that one can come to deserve accolades and fame by winning a gold medal at the Olympics, though this achievement is arguably morally neutral, or at least, not very morally significant.

Limiting the scope of the thesis to achievements whose realization contributes positively to the achiever’s desert has the advantage of leaving open two quite different ways in which the desert thesis could turn out to be correct. It could be that employing enhancements generally diminishes the positive influence of realizing an achievement on one’s desert, or it could be that employing enhancements generally makes an independent negative contribution to desert.

## Motivating the desert thesis

Having clarified the desert thesis, I am now in a position to explain why I take it as my focus. The thesis has at least three claims to philosophical attention.

First, it yields intuitively plausible verdicts about desert in many cases. Consider what is perhaps the paradigmatic example of enhancement: doping in sport. When one athlete succeeds with the assistance of a doping substance, we would generally intuit that this athlete deserves the rewards associated with his success less than other similarly successful athletes who do not dope. Similar thoughts apply to the use of cognition enhancement drugs by students during examinations. Consider the case of Acheson and Baird, with which I began. Most would judge that Acheson, who used a cognition-enhancing drug, deserves her place on the prestigious PhD programme less than Baird, who did not. This is precisely what the desert thesis would suggest.

Second, the desert thesis, or a restricted version of it, appears to be held, at least implicitly, by some members of the public in social-psychological research. Faber and collaborators presented healthy participants with a scenario in which a protagonist succeeds in completing a cognitively demanding task either with or without the assistance of a cognition-enhancing drug. Participants attributed the success less to the protagonist and deemed the success to be less deserved, when the protagonist had used a cognitive enhancer than when she had not (Faber et al., 2015; Faber et al., 2016). The research subjects appeared to be invoking something like the desert thesis, at least in relation to enhancements of cognition.

Third, though the desert thesis has, to my knowledge, never been straightforwardly asserted in the philosophical literature on enhancement,^3^ it is charitable to attribute it to some of those who advance moral or prudential objections to enhancement.

For instance, certain fairness-based objections to enhancement are difficult to sustain without it. Many have objected to enhancements on the ground that they produce unfairness (Douglas, 2007; Fukuyama, 2002: 9–10, 97; Gazzaniga, 2006; President’s Council on Bioethics, 2003: 131–134, 280–281; Rose, 2006: 303; Savulescu, 2006; Schermer, 2008b). In some cases, the concern is about unfair inequalities that might result from unequal access to enhancement technologies or about straightforward cheating – the breaking of rules or conventions that others respect. However, others worry that enhancements will generally (*viz.*, always or almost always) produce unfairness *regardless* whether the non-enhancers lacked access to enhancements or simply chose not to use them, and regardless whether the enhancement infringes any law or convention (Schermer, 2008b).

The desert thesis can, when conjoined with some prominent accounts of fairness, explain why enhancement will generally produce unfairness even in such cases. The relevant accounts of fairness hold that fairness, or one species of it, requires that goods are distributed across individuals in proportion to the degree to which those individuals deserve those goods (Feldman, 1995a; Kagan, 2012: 351).^4^ Thus, it is unfair if I have twice as much of some good as you though we are equally deserving of it: fairness then requires that, if possible, we enjoy the same amount of this good. It is also unfair if I have the same amount of that good as you though you deserve it more: fairness then requires that, if possible, you enjoy more of that good.^5^

Suppose that you and I succeed in the same way and enjoy the same rewards as a result. But suppose that I realized that success with the aid of an enhancement, whereas you did not. In all other respects, we are similar. For example, we were, in advance of realizing the achievement, equally well off and equally deserving of the rewards we both go on to receive. If the desert thesis holds, then, having both realized the achievement, you are more deserving than me, and it is unfair that we in fact enjoy the same rewards; fairness requires that, if possible, you enjoy greater rewards than me. By contrast, had I realized the achievement without the enhancement or simply not realized the achievement, there is no reason to suppose that any unfairness would have been present between us. My use of the enhancement has had a fairness-disrupting effect.

This is not to say that my use of the enhancement has caused an increase in unfairness *all told*, for my use of the enhancement may also mitigate some existing unfairness. Suppose, for example, that the rewards that you and I acquire through our achievements consist in some quantity of good *G.* And suppose that, besides you and me, everyone enjoys some very large quantity of *G* – much more than we enjoy before, or indeed even after, our achievements. Suppose, moreover, that these others are in fact no more deserving of their large endowments of *G* than us. We thus possess smaller endowments of *G* than fairness requires. In this setting, even though my use of an enhancement may introduce an unfairness between you and me, it will also reduce the unfairness between me and everyone else by reducing the degree to which I am under-endowed with *G*, relative to them.^6^ All things considered, the situation may have improved with regard to fairness. Still, my use of the enhancement has had *some* fairness-disrupting effect; it introduces unfairness between you and me.

The desert thesis also helps to make sense of another oft-heard objection to enhancement. Opponents of enhancement sometimes invoke the claim that achievements accomplished with the aid of enhancements lack (some of) the value normally attached to achievements of the same kind. For instance, Eric Juengst wonders whether achievements realized via enhancement might be ‘hollow accomplishments’, while the President’s Council on Bioethics claimed that enhancements would undermine the ‘dignity’ of human performance and perhaps render that performance ‘false’ (Juengst, 2000: 39; President’s Council on Bioethics, 2003: 131, 140).

In some cases, enhancements may devalue our achievements because they frustrate the very purpose of the activity being pursued; using an enhancement might, to take an oft-cited example, be like completing a marathon with the aid of roller skates (Whitehouse et al., 1997: 18). Some activities – like marathon running – fulfil their purpose only where pursued in a certain kind of way, and, in some cases, enhancement is inimical to the required manner of execution.

However, not all activities are such that their purpose is undermined when they are pursued with the aid of enhancements (Douglas, 2007; Mehlman, 2004: 493; Roache, 2008; Santoni de Sio et al., 2014; Schermer, 2008a, 2008b). Consider landing an airliner or performing a surgical operation. The purpose of these activities is to realize a certain outcome, and the realization of that outcome need not be threatened, and may even be aided, by the use of enhancements. Nevertheless, some present the concern about devaluing achievements as a *general* worry about enhancement (President’s Council on Bioethics, 2003: 140–150).

The desert thesis is able to explain why enhancement might deprive an achievement of some of its value even if the activity being pursued by the enhanced individual continues to serve its valuable purpose. It is often thought that normally valuable evaluenda can lack their normal value when they are not deserved (Brentano, 1969: 149; Feldman, 1995c).^7^ Pleasure is normally valuable, but some would maintain that it lacks some or all of its normal value when it is not deserved. It is plausible that similar thoughts apply to the goods associated with achievements. It may be that the value of these goods diminishes as the degree to which they are deserved decreases. Thus, if enhancements undermine desert in the way the desert thesis claims, we can expect the achievements that they enable to lack some of their normal value, as proponents of the present objection maintain.

I believe that it is charitable to impute the desert thesis to many of those who have claimed that enhancement generally produces unfairness or devalues our achievements. However, even if this is not so, the fact that the desert thesis provides one plausible way of supporting those widely advanced claims gives us some reason to at least entertain it as an ethical hypothesis. Let us now turn to testing that hypothesis, which I propose to do by examining three candidate justifications for it. The first of these is what I call the argument from immoral means.

## The argument from immoral means

Many believe that enhancement is always morally undesirable in a way that education, training and the mere exertion of effort are not. I have suggested that some of this opposition is grounded on the desert thesis. But some is clearly not. Some believe that enhancements are undesirable for independent reasons, for example, because they alienate the enhanced individuals from their true selves (Elliott, 2004; Glannon, 2001: 81–82; Parens, 2005; President’s Council on Bioethics, 2003: 252–260, 293–295), restrict their freedom or autonomy (Habermas, 2003: 64–90), express an ugly desire for mastery (President’s Council on Bioethics, 2003: 287–290; Sandel, 2007: 46, 85), undermine social solidarity (President’s Council on Bioethics, 2003: 56; Sandel, 2007: 89–91) or poison healthy family and romantic relationships (Parens, 2005; President’s Council on Bioethics, 2003: 50–51, 54–55; Sandel, 2007: 45–62). Let us suppose, for the sake of argument, that at least one of these criticisms is sound – that enhancements are always morally undesirable in some desert-independent way that education, training and the mere exertion of effort are not. In this case, we will have a ready justification for the desert thesis, for it is plausible that the adoption of morally undesirable means tends to diminish one’s desert.^8^

Thus, suppose that Ewart wins Olympic gold in figure skating, but he wins only because he previously assaulted and seriously injured one of his competitors. Or suppose that Ferguson wins a Nobel Prize in Medicine, but she wins only because she ruthlessly exploited her junior staff. Intuitively, these individuals deserve the goods associated with their success less than the typical Olympic gold medallist or Nobel Prizewinner, and one explanation for this appeals to the fact that each realizes the achievements through morally undesirable means. If the employment of enhancements is always in one way morally undesirable, then similar reasoning could be applied to achievements realized with the assistance of enhancements.

The difficulty with this approach to defending the desert thesis is that it relies on the existence of an independent (of desert) basis for supposing that enhancements are morally undesirable. To establish that any *particular* enhancement undermines desert, we must first establish that there is an independent objection to it, and to establish that enhancement *generally* undermines desert, as the desert thesis maintains, we must establish that there is an independent general objection to enhancement. This deprives appeals to desert of much of their dialectic interest in the ethical debate on enhancement. Such appeals derive much of their interest from their potential to fill a justificatory gap – to establish that enhancements are morally or prudentially undesirable in some cases where we would otherwise be unable to establish this. On the present argument, however, appeals to desert can at most establish a supplementary objection to enhancement.

Let us turn, then, to consider whether it is possible to defend the desert thesis in a way that does not diminish its dialectic significance in this way.

## The argument from diminished effort

A second argument for the desert thesis is suggested by the claim, sometimes advanced as an objection to enhancement, that enhancements make our achievements ‘too easy’. Ronald Cole-Turner worries that

The new means [of augmenting normal human capacities], such as psychopharmacology and genetic alteration, are perhaps more efficient or easier than the traditional means. But for that reason, they should be opposed…There is, after all, a glory and a dignity in human accomplishment attained the “old-fashioned way,” through sweat and struggle, sometimes against great odds. (Cole-Turner, 2000: 155)

Leon Kass develops the point further:

in those areas of human life in which excellence has until now been achieved only by discipline and effort, the attainment of those achievements by means of drugs, genetic engineering, or implanted devices looks to be “cheating” or “cheap.” We believe – or until only yesterday believed – that people should work hard for their achievements. “Nothing good comes easily.” Even if one prefers the grace of the natural athlete, whose performance deceptively appears to be effortless, we admire those who overcome obstacles and struggle to try to achieve the excellence of the former….(Kass, 2003: 21. See also Sandel, 2007: 25–26; Tobey, 2003: 113–114, 120–124).^9^

One way of making sense of these worries would see them as adverting to the putative relationship between effort and desert. It is often thought that the exertion of effort can confer desert (Milne, 1986; Sadurski, 1985; Sorensen, 2010), and it is not difficult to generate intuitions that support this view. Suppose that Gibbs and Hamilton both complete a marathon in slightly less than 4 hours. The two trained similarly hard for the event, but there is a large gulf in natural ability between the two. Gibbs is a naturally gifted runner, so for him, completing the marathon in less than 4 hours took little effort. Indeed, he has run much more quickly in the past. Hamilton, by contrast, is genetically unsuited to long-distance running. For him, completing the marathon at all took Herculean effort.

It would be tempting to say, regarding this case, that Hamilton is in one way more deserving of the goods associated with his achievement than Gibbs. This is not to say that he is all-things-considered more deserving – some would argue that, perhaps since sports are designed in part to test or display natural ability, one of the things that can make one deserving of sporting success, and associated rewards, is precisely the kind of natural ability that Gibbs possesses. But there seems to be something pulling in the other direction also. There seems to be some respect in which Hamilton *is* more deserving than Gibbs. A natural way to account for this intuitive judgment would be to appeal to the fact that Hamilton must have exerted more effort than Gibbs to achieve his success.

Consider now a second case, this time involving the use of a (let us suppose) permitted enhancement. Irvine and Jardine have roughly equal innate running ability and have trained equally hard. They both complete a marathon in just less than 3 hours – much faster than either has run previously. Irvine’s unusually quick time is due to her pushing herself exceptionally hard during the race; Jardine’s is attributable to her having taken a blood-thickening dietary supplement in the weeks leading up to the race. Again, we might be tempted to say that Irvine deserves any success associated with her achievement more than does Jardine. And again, we might posit that this is due to Irvine’s greater exertion of effort.

We can generalize from this case. It is not a coincidence that Jardine employed an enhancement *and* exerted less effort than Irvine. It might seem that, *in general*, individuals who have employed enhancements will have exerted less effort to realize an achievement than other individuals who realize the same achievement without the assistance of enhancements but instead through training, education or merely trying harder. All of these alternative routes to achievement typically involve greater exertion of effort than does the pursuit of enhancement.^10^ Perhaps, then, enhanced achievers are generally less deserving than comparable unenhanced achievers because they generally exerted less effort in the realization of those achievements.

This argument – the argument from diminished effort – may seem susceptible to a quick response: in some cases, enhancements lead the enhanced individual to exert *more* effort than she would otherwise have exerted. Indeed, some enhancements work precisely by augmenting a person’s ability or willingness to exert effort; consider the use of anabolic steroids to speed muscle recovery after training, thus allowing an athlete to train again more quickly. Even if the *pursuit* of an enhancement requires less effort than alternative means of augmenting one’s abilities, one *effect* of the enhancement may be to increase one’s exertion of effort, and this desert-augmenting influence could predominate.

I believe that this quick response may be sufficient to undermine the argument from diminished effort, but I will not pursue it here, for two reasons.

First, it applies only to a rather narrow range of enhancements. This response may show that *some* enhancements augment effort and thus desert, and thus undermine the desert thesis, as I have characterized it. However, the victory may end up being rather hollow, for the great majority of enhancements may nevertheless undermine effort, and thus desert. By contrast, the response that I will offer below applies more generally.

Second, the quick response is susceptible to a possible objection. The objection holds that what matters for desert is not the absolute level of effort that one exerts, but the level of effort one exerts relative to one’s potential for exerting effort. What matters, in other words, is the degree to which one realizes this potential. If ‘potential for exerting effort’ is understood so as to incorporate all biological influences on the level of effort that one in fact exerts, then enhancements, whose effects are biological, must operate by augmenting potential for exerting effort, rather than the degree to which this potential is realized. On the present objection, this will confer no increase in desert.

I do not wish to endorse this objection – indeed, I think its prospects are not rosy, for reasons I have pursued elsewhere (Douglas, 2014a). Still, if we could offer a response to the argument from diminished effort that evaded this sort of objection, that would be preferable, and as it happens, I believe that we can do just that.

Rather than employing the quick response, which holds that some enhancements augment effort, I will pursue an alternative line of response to the argument from diminished effort. My response concedes that enhancements undermine effort, but maintains that they nevertheless need not undermine desert.

The response begins from the observation that, in some circumstances, adopting a lower effort route to some achievement has no negative effect on one’s desert for the goods associated with that achievement (Schermer, 2008a: 359–360). One such circumstance is where an agent has the option of realizing the achievement via either a lower or a higher effort route, and there is no more reason to pursue the higher effort route than the lower effort route. In such a case, adopting the higher effort route involves exerting gratuitous effort – effort that there is no more reason to exert than not – and it would be at the very least puzzling if gratuitously doing things the hard way could make one more deserving of goods (Douglas, 2014b; Milne, 1986: 240).^11^

Suppose, then, that two similar agents face a choice regarding how to realize some achievement. They can either employ an enhancement or a more effortful traditional route involving, say, extra training. Both options are available to both agents, and the agents are aware of the amount of effort that each route to the achievement entails. The first agent chooses to employ the enhancement; the second adopts the more traditional route.

If there is no more reason to abstain from the enhancement than to pursue it, then the second agent, who eschews the enhancement, is simply exerting gratuitous effort, and exerting gratuitous effort cannot make one more deserving.^12^

Of course, in many circumstances in which enhancements are used, there may be reasons to adopt a higher effort route. For example, if we think that part of the point of sports is to elicit extreme levels of effort, then the athlete may well have reasons to eschew effort-reducing enhancements. However, if this is the case, we already have an independent (of desert) objection to pursuit of the enhancement. Thus, we are back with the problem faced by the argument from immoral means. The appeal to diminished effort will succeed in establishing that a *particular* enhancement undermines desert only if there is an independent objection to that enhancement, and it will succeed in establishing the desert thesis only if there is an independent *general* objection to enhancement. Without an independent objection to an enhancement, there will be no reason to suppose that we should prefer traditional routes to the realization of an achievement – no reason to suppose that the additional effort exerted in the pursuit of the traditional route would be nongratuitous.

Two possible responses to this suggestion need to be considered. The first maintains that the effort exerted in avoiding an enhancement is rarely gratuitous, because effort almost always has the instrumental value of helping to cultivate a disposition or ability to exert (nongratuitous) effort in the future. Exerting effort, it might be said, is generally character-building. Yet even if exerting effort did generally conduce to desirable forms of psychological development, a claim that has been questioned (Forsberg, 2013: 168–169; Schermer, 2008a: 357–358), there is no good basis for assuming that reasons to pursue such psychological development will always, or even typically, be decisive. After all, exerting effort also has predictable costs. Realizing a given achievement via a more rather than a less effortful route will, for example, tend to diminish one’s ability and willingness to realize other valuable goals.

The second response maintains that even gratuitous effort makes one more deserving. Certain intuitive reactions might seem to support this view. Suppose that Kerr and Lester independently make the same scientific discovery. Both relied on the same sophisticated piece of statistical software in making this discovery, however, as an additional challenge, Kerr had one of her colleagues program a number of bugs into the software. Thus, Kerr had to overcome a number of software glitches to make the discovery, whereas Lester did not. Kerr had to exert more effort. Suppose, moreover, that any reason Kerr had to exert this effort in order to develop desirable character traits was outweighed by countervailing reasons: perhaps, for example, the effort of overcoming the software bugs also drained her energy in a way that predictably impaired her ability to produce other smaller scientific insights in the course of making her chief discovery.

Although I do not share the intuition myself, I concede that some might wish to claim that Kerr deserves more praise for her achievement than Lester, even though the extra effort she exerted appears to be gratuitous.

It is not clear, however, that this intuition supports the view that gratuitous effort can confer desert, for we can accommodate the intuition without endorsing that view. We may take Kerr to be more deserving of praise than Lester because we take her exertion of gratuitous effort as evidence that had a greater level of effort been necessary to realize the achievement – say, because only buggy software was available – she would have exerted it. This would suggest that it is the counterfactual exertion of nongratuitous effort by Kerr that confers her greater desert, not her actual exertion of gratuitous effort (Douglas, 2014b).

Thus, it is possible to explain the intuition that Kerr is more deserving than Lester without attributing a desert-conferring effect to gratuitous effort, and since it would be at the very least puzzling if gratuitous effort could confer desert, we should tentatively accept this alternative explanation.

I take it, then, that exerting gratuitous effort confers no desert. It follows that, for any given enhancement, there is either an independent (of desert) objection to the pursuit of the enhancement, thus deflating the dialectic significance of the claim that the enhancement undermines desert, or the appeal to diminished effort fails to establish that the enhancement undermines desert, since the effort exerted through eschewing the enhancement would have been nongratuitous.

## The argument from shared responsibility

A third argument for the desert thesis is suggested by another claim sometimes advanced as an objection to enhancement. Daniel Tobey writes that

With enhancement we achieve an end not through struggle but with the ease of one selecting an item from a menu – it is the difference between cooking a meal and ordering in. The purchased meal might taste better objectively, but it is not *ours*, and in not working for it, we lose the sense of ownership and accomplishment. (Tobey, 2003: 121)[original emphasis]

The primary claim here is ostensibly that enhancement might diminish our *sense of* ownership for our achievements, but Tobey also appears to suggest that we would *in fact* own our achievements less, for he writes that the purchased meal ‘*is* not ours’ [my italics]. I take Tobey to be suggesting here that enhanced individuals are not responsible (or not fully responsible) for the achievements that their enhancements enable (Sandel, 2007: 26).^13^

Responsibility should, for the purposes of this discussion, be understood as attributability (Watson, 1996).^14^ An agent *A* is in this sense responsible for *x* if and only if *x* can aptly be attributed to agent *A.* Attributability is more than mere causal responsibility, since it necessarily involves agency on the part of the responsible individual; indeed, it is normally supposed that *x* is attributable to *A because x* is a product or expression of *A*’s agency. On the other hand, attributability is less than (though it is probably necessary for) moral responsibility, where the latter is taken to entail moral praise- or blame-worthiness. An action could be an expression of one’s agency, and thus attributable to one, though the agency it expresses is morally unremarkable and one is thus worthy of neither praise nor blame (Levy, 2005).

I take it, then, that Tobey is suggesting that use of enhancements generally diminishes the degree to which the resulting achievements can be attributed to the agent who undergoes the enhancement. Why think that enhancement diminishes responsibility in this way?

One thought might be that the responsibility of enhanced achievers is diminished because some responsibility for the achievements that they realize lies elsewhere – it lies with the designers (and perhaps also the prescribers) of the enhancement. A parallel point can be made regarding the use of external technologies in certain sports: Consider the yachtswoman who wins a regatta because her friend loans her an especially fast boat, or the Formula One driver who wins a grand prix because his team provides him with the fastest car available. We might be inclined to say that these competitors are less responsible for their victories than they would have been had they realized them with a standard yacht and car, respectively, because more of the responsibility for their achievements is borne by others: the designers and providers of the assistive technologies. The proponent of the present claim takes a similar view about achievements enabled by enhancements: The responsibility of the enhanced achiever is reduced because that responsibility is shared with, for example, the designers and distributors of the enhancement technology.

If the partial responsibility of others generally diminishes the responsibility of enhanced achievers for the achievements they realize, then we will, I think, have a plausible argument for the desert thesis. Sometimes, realizing an achievement confers desert in the sense that, through achieving something, the achiever comes to deserve (some of) the goods associated with the achievement, or to deserve them to a greater degree. As noted earlier, this is not always so: for example, realizing *evil* achievements does not make one (more) worthy of any associated rewards. But we are limiting ourselves to cases in which realizing an achievement without the aid of an enhancement would make one more deserving of the associated goods. In such cases, we might expect that the degree of desert conferred will tend to diminish with one’s responsibility for the achievement. A reduction in the degree to which one is responsible for an achievement will tend to reduce the degree to which one deserves the goods attached to that achievement.^15^ This meshes well with our intuitions about the yachtswoman and Formula One driver. Intuitively, these individuals are not only less responsible for their victories than they would have been had they achieved them without superior equipment, they are also less deserving of the rewards that come with these victories, even if they remain fully entitled to those rewards, for instance, because the rules of the competition allow them to use such superior equipment.

If the use of enhancements generally reduces one’s responsibility for one’s achievements, then there is a *prima facie* credible argument for the desert thesis. However, it is far from clear that enhancement does reduce responsibility in this way. Intuitions about the yachtswoman and Formula One driver might seem to support this view. But two cases cannot prove the rule. These cases show, at most, that *in some instances* reliance on technology diminishes responsibility, but what we need is a justification for the view that that reliance on technology, or at least enhancements, will *generally* (that is, always or almost always) have this effect.

Let us consider two candidate justifications. The first holds that responsibility is zero-sum: for any achievement, there is a fixed amount of responsibility to go around. Thus, if the designer of an enhancement is partly responsible for a post-enhancement achievement, the user of the enhancement is necessarily not fully responsible for it.

This justification has unacceptable implications. Suppose that *A, B* and *C* together rob a corner store, while elsewhere and entirely independently *D* robs a different corner store. Each of *A*, *B, C* and *D* is, then, partially or fully responsible for the robbing of a corner store. But intuitively, the degree of responsibility of each of *A, B* and *C* for the robbing of a store is more than a third of the degree of responsibility of *D*. Consequently, we would not, for example, be inclined to punish each of *A, B* and *C* only a third as severely as *D*. The total degree of responsibility of *A, B* and *C* is greater than that of *D*, indicating that responsibility for the robbing of a store is not zero-sum; the total amount of responsibility can increase as the number of individuals who share the responsibility increases.

The claim that responsibility is zero-sum has implausible implications. Let us turn, then, to the second candidate justification for the view that the use of enhancements reduces responsibility. This justification holds that, when you use an enhancement to achieve some outcome, you ‘outsource’ some of the agential processes required to bring about that outcome; you employ someone else to invest her agency in realizing the achievement, and in doing so you diminish the role played by your own agency. Thus, the achievement is less an expression of your agency than it would otherwise have been. When the Formula One driver accepts the faster car from his team, it is not just that he allows the agency of others to influence his level achievement, he reduces the call for investing his own agency. Without the faster car, he would have had to train harder, or make better tactical decisions, to achieve the same result. Perhaps those who realize achievements with the aid of enhancements can also be thought of as outsourcing some of the agential tasks required to realize an achievement.

This justification – the ‘outsourcing justification’ – also runs into difficulties, however.

An initial response to it would mirror the ‘quick response’ to the argument from diminished effort that I considered in the previous section. Even if outsourcing agential tasks would in one way tend to diminish the role played by the enhanced person’s own agency in realizing an achievement, it might in other ways tend to augment her investment of agency: for example, the enhancement may operate by augmenting the agential resources that the enhancement user has available to invest (Santoni de Sio et al., 2016). This will most obviously be so in relation to enhancements that diminish psychological impediments to the exercise of agency. Suppose you face a choice between several alternative courses of action, but suppose you have a number of false beliefs about the nature and likely consequences of these options. Suppose also that you are subject to a number of impulses that might lead you to take an option other than the one you reflectively endorse. These false beliefs and impulses are plausibly impediments to your agency. But a cognition enhancement might help to correct these false beliefs, and a motivation enhancement might attenuate these impulses. A possible upshot of either enhancement would be that your decision becomes more reflective of your agency and less reflective of impediments to it.

As with the quick response to the argument from diminished effort, I believe that this response may be sufficient to refute the outsourcing justification, and thus, the argument from shared responsibility, but, also as with that response, it will apply to a rather narrow range of enhancements, and it may be susceptible to the objection that what matters is not absolute investment of agency, but investment relative to one’s potential to do so. For these reasons, I pursue an alternative response to the outsourcing justification – one that applies more generally and is not susceptible to this possible objection.

My response begins from the observation that, on the outsourcing justification, it is not ultimately the enhanced individual’s increased reliance on others’ agency that diminishes her responsibility, it is her decreased reliance on her own agency. On the outsourcing justification, then, cases of enhancement are relevantly similar to cases in which a person is less reliant on her own agency not because she has deployed an enhancement, or otherwise outsourced a task to someone else, but for some other reason: for example, through good fortune of circumstance. The scientist who employs a cognitive enhancement and thus diminishes the need to exert her own agency in her work is, from the perspective of the outsourcing justification, equivalent to a scientist who, through no good planning of her own, happens to find herself in an inspiring and distraction-free environment; an environment that allows her to make scientific progress with less need to invest her own agency than a comparable scientist in less favourable conditions. Note, however, that we would not normally regard an individual as less responsible for her achievements, in the sense that the achievements are less attributable to her, merely because she, though good fortune, finds herself in more favourable conditions and thus with less need to invest her own agency.

There are at least two plausible explanations for why reduced investment of agency does not necessarily reduce responsibility. On the first explanation, full responsibility is retained as long as the investment of agency surpasses some threshold. Thus, there is a range over which a reduction in agency invested confers no reduction in responsibility. On the second explanation, only a certain *kind* of agency needs to be invested in order to fully preserve responsibility. For example, it may be necessary only that the individual exercises executive control over how her abilities, the assistance of others, and any other available resources are marshalled towards the realization of an achievement. On this view, an individual can outsource many individual tasks required for the realization of an achievement without diminishing her own responsibility, provided that she continues to exercise executive control in drawing on the assistance of others. Thus, for example, if the Formula One driver in our above-mentioned example had been actively involved in deciding which technological innovations in car design to adopt, his responsibility for his grand prix victory would not have been diminished by his being provided with the especially fast car.

Both of these explanations for why reduced investment of agency need not reduce responsibility could be invoked in relation to some enhancements. Even where enhancements involve outsourcing tasks to others, thereby reducing the need for the enhanced individual to invest his own agency, they may nevertheless leave the enhanced individual above the threshold level of agency-investment required for full responsibility on the first explanation. And provided that the enhancer continues to exercise his agency in, for example, deciding how to employ enhancements, or the abilities that they confer, he may retain the kind of executive control required for full responsibility according to the second explanation.

Reflection on cases in which the call for agential investment is diminished by accidents of circumstance suggests that the cases in which enhancements diminish responsibility by diminishing agential investment will be rare. In most cases, those who, through good fortune, enjoy extremely favourable circumstances nevertheless retain full responsibility, in the sense of attributability, for their achievements, suggesting that only a modest level of agential investment or executive control is required for full responsibility. This suggests that most enhancements will also preserve full responsibility.

## Implications

I have assessed three arguments for the desert thesis. I argued that the third of these – the argument from shared responsibility – fails to justify the thesis: It is not the case that enhancements generally diminish responsibility; indeed, I have just suggested that in most cases they do not. By contrast, I allowed that the first two arguments – the argument from immoral means and the argument from effort – might succeed. However, I argued that they succeed in showing that an enhancement undermines desert only in those cases where there is already an independent objection to the enhancement, and in these cases, the appeal to desert loses much of its dialectic interest.

It is possible, of course, that there is some other argument for the desert thesis that I have missed. However, it is not clear what this argument could be, and the literature on the ethics of enhancement is of little help here.^16^ In that literature, the desert thesis typically functions as a premise (usually implicit), not as a conclusion, and when authors sympathetic to the desert thesis have alluded to considerations that might support it, they have alluded to the argument from diminished effort and the argument from shared responsibility.

Let us thus tentatively assume that we have assessed the only credible arguments for the desert thesis. This leaves the proponent of the thesis with two options. First, she can rely on the argument from shared responsibility and accept that there is no rational basis for the view that enhancement generally undermines desert, or indeed for the view that it does so in any more than a very narrow range of cases: Most enhancements preserve full responsibility. Second, she can turn to either the argument from immoral means or the argument from diminished effort. However, she is then forced to concede that the desert thesis either fails (because there is no general independent objection to enhancement) or has little dialectic significance (because there is such an objection). Moreover, she is then forced to concede that desert-based explanations for why any *particular* enhancement is morally problematic will either fail or have little dialectic significance.

Nevertheless, my argument does draw attention to some cases in which use of enhancements will undermine desert. One of these cases is where the enhancement dramatically diminishes the investment of agency on the part of the enhanced individual. Another is where the enhancement reduces the amount of effort that the enhanced individual exerts but there were, in fact, good independent reasons to exert that effort. One such case will be where the enhancement is employed in the context of an activity whose value lies, at least in part, in its effortfulness (which I suspect is true of many sporting endeavours); in such contexts, there *is* an independent objection to avoiding effort, and thus to pursuing the enhancement: doing so undermines the value of the activity in which one is engaged.

## Notes

1. I henceforth use ‘generally’ as shorthand for ‘always or almost always’.

2. Note that all of this is consistent with there being some factors that contribute to or detract from desert across all institutional contexts. It is also consistent with worthiness and entitlement coming apart. For example, suppose that conventions require that a sporting prize is given to the sporting competitor who performs the best on the day, even though the (conventionally determined) purpose of the sport is to identify the person with the greatest sporting ability and that person may not perform the best on the day. In this context, it is plausible that a more able competitor is *ceteris paribus* more worthy than a less able competitor who outperforms him on the day.

3. Some have come close to asserting the desert thesis. For example, the President’s Council of Bioethics suggested that performance enabled through enhancement might be ‘less worthy of admiration’ than other kinds of performance (President’s Council on Bioethics, 2003: 140). Variants of the thesis are also briefly critically discussed by Mehlman (2004: 492–493) and Schermer (2008a).

4. Note, however, that neither of these authors describes their positions precisely as I have here. Kagan prefers to use the term ‘comparative desert’ in place of what I call ‘fairness’, though he acknowledges that the term ‘desert sensitive fairness’ could also be used (2012: 351); Feldman’s view invokes not fairness, but the closely related concept of justice. John Broome has offered a similar but broader notion of fairness according to which fairness requires distribution according to the moral claims of individuals, where desert is only one among several considerations that could give one a moral claim (Broome, 1990).

5. I assume here that the good is divisible. I remain silent on precisely what distribution of rewards is proportionate to desert in cases where individuals differ in their degrees of desert. For instance, I remain silent on whether fairness requires that *A* enjoys precisely twice as much of a good as *B* if *A* is twice as deserving of it.

6. It reduces this under-endowment by increasing my endowment of *G*, and perhaps also by reducing my desert for *G*.

7. Note that Brentano and Feldman do not deny that undeserved goods remain good for the possessor of those goods. The claim is that they do not have their usual value for the world.

8. I assume here that the moral desirability of the means via which individuals pursue their goals is always known to them in advance.

9. For critical discussion, see Schermer (2008a: 359–360); and Buchanan (2011: 110–112).

10. It is not clear that augmenting one’s abilities via enhancement *always* requires no effort or even less effort than augmenting them via more traditional means (see, for example, Mehlman, 2004: 492). Imagine a surgeon who has a strong aversion to taking an attention-enhancing drug, but judges that she really ought to take one, since she believes this will help to protect her patients from surgical errors. This surgeon may find that it takes considerable effort to override her initial aversion and take the drug. It may take as much or even more effort for her to undergo the enhancement as it would to bring about an improvement in her attention through some alternative, more traditional means. Nevertheless, I will assume for the sake of argument that enhancements always require less effort than alternative means of augmenting one’s abilities.

11. It might be argued that what matters is not whether the effort one exerts is gratuitous, but whether it is (reasonably) believed to be gratuitous. Suppose you exert gratuitous effort to realize some achievement, but you (reasonably) believe it to be nongratuitous. Then it might seem that this effort could make you more deserving, since it was at least well-intended. However, this will not help the proponent of the desert thesis, since if what matters is whether effort is (reasonably) believed to be gratuitous, then the argument from diminished effort will fail whenever the effort involved in avoiding an enhancement is (reasonably) believed to be gratuitous.

12. It might still be argued that the individual who uses the enhancement is less deserving than one who only has the higher effort route available – someone who has no access to enhancement.

13. For critical discussion, see Goodman (2010: 155–157); Buchanan (2011: 110–112); and Forsberg (2013).

14. A variant of responsibility-as-attributability that applies only to the outcomes of actions (but is otherwise, I believe, co-intensive with the more general concept) has played a prominent role in the theory of tort law, where it is normally described as ‘outcome responsibility’ (Honoré, 1988; Perry, 1992: 497–498).

15. A number of philosophers have defended claims about the relationship between responsibility and desert that would support this view (Rachels, 1978: 157; Sadurski, 1985: 117, 131; Smilansky, 1996). For criticism, see Feldman (1995b) and Cupit (1996).

16. Santoni de Sio and collaborators offer an explanation for why enhanced individuals deserve less praise than unenhanced individuals *in some cases*: using an enhancement, individuals change the nature of the activity they are engaged in, and in such a way that a new yardstick for evaluation must now be used (Santoni de Sio et al., 2016). As the authors note, however, this argument applies only to certain kinds of activity: those partly constituted by rules which the use of enhancement would violate.

## References

[bibr1-1470594X18810439] Brentano F (1969) Loving and Hating, the Origin of Our Knowledge of Right and Wrong, Kraus O (ed.) Chisholm R Chisholm R Schneewind E (transl.). London: Routledge.

[bibr2-1470594X18810439] Broome J (1990) Fairness. Proceedings of the Aristotelian Society 91: 87–102.

[bibr3-1470594X18810439] Buchanan AE (2011) Beyond Humanity?: The Ethics of Biomedical Enhancement. Oxford: Oxford University Press.

[bibr4-1470594X18810439] Cole-Turner R (2000) Do means matter In: Parens E (ed.), Enhancing Human Traits: Ethical and Social Implications. Washington: Georgetown University Press, pp. 151–161.

[bibr5-1470594X18810439] Cummiskey D (1987) Desert and entitlement: a Rawlsian consequentialist account. Analysis 47: 15–19.

[bibr6-1470594X18810439] Cupit G (1996) Desert and responsibility. Canadian Journal of Philosophy 26: 83–99.

[bibr7-1470594X18810439] Douglas T (2007) Enhancement in sport, and enhancement outside sport. Studies in Ethics, Law, and Technology 1. 10.2202/1941-6008.1000 PMC2742076 19750128

[bibr8-1470594X18810439] Douglas T (2014a) Enhancing moral conformity and enhancing moral worth. Neuroethics 7: 75–91. 24600486 10.1007/s12152-013-9183-y PMC3933743

[bibr9-1470594X18810439] Douglas T (2014b) The relationship between effort and moral worth: three amendments to Sorensen’s model. Ethical Theory and Moral Practice 17: 325–334. 24991189 10.1007/s10677-013-9441-4 PMC4074890

[bibr10-1470594X18810439] Elliott C (2004) Better Than Well: American Medicine Meets the American Dream. New York: W. W. Norton & Company.

[bibr47-1470594X18810439] Faber NS Douglas T Heise F Hewstone M (2015) Cognitive Enhancement and Motivation Enhancement: An Empirical Comparison of Intuitive Judgments. AJOB Neuroscience 6: 18–20.

[bibr11-1470594X18810439] Faber NS Savulescu J Douglas T (2016) Why is cognitive enhancement deemed unacceptable? The role of fairness, deservingness, and hollow achievements. Frontiers in Psychology 7: 232. 26925027 10.3389/fpsyg.2016.00232 PMC4759582

[bibr12-1470594X18810439] Feinberg J (1970) Doing and Deserving: Essays in the Theory of Responsibility. Princeton: Princeton University Press.

[bibr13-1470594X18810439] Feldman F (1995a) Adjusting utility for justice: a consequentialist reply to the objection from justice. Philosophy and Phenomenological Research 55: 567–585.

[bibr14-1470594X18810439] Feldman F (1995b) Desert: reconsideration of some received wisdom. Mind 104: 63–77.

[bibr15-1470594X18810439] Feldman F (1995c) Justice, desert, and the repugnant conclusion. Utilitas 7: 189–206.

[bibr16-1470594X18810439] Forsberg L (2013) No Pain, no gain? Objections to the use of cognitive enhancement on the basis of its potential effects on the value of achievement In: Hildt E Franke AG (eds), Cognitive Enhancement: An Interdisciplinary Perspective. Dordrecht: Springer Netherlands, pp. 159–171.

[bibr17-1470594X18810439] Fukuyama F (2002) Our Posthuman Future: Consequences of the Biotechnology Revolution. New York: Profile Books.

[bibr18-1470594X18810439] Gazzaniga M (2006) The Ethical Brain: The Science of Our Moral Dilemmas, reprint edition. New York: Harper Perennial.

[bibr19-1470594X18810439] Glannon W (2001) Genes and Future People: Philosophical Issues in Human Genetics. Boulder: Westview Press.

[bibr20-1470594X18810439] Goodman R (2010) Cognitive enhancement, cheating, and accomplishment. Kennedy Institute of Ethics Journal 20: 145–160. 20653250 10.1353/ken.0.0309

[bibr21-1470594X18810439] Habermas J (2003) The Future of Human Nature. Maldon: Polity Press.

[bibr22-1470594X18810439] Honoré T (1988) Responsibility and luck: the moral basis of strict liability. Law Quarterly Review 104: 530–553.

[bibr23-1470594X18810439] Juengst E (2000) What does enhancement mean? In: Parens E (ed.), Enhancing Human Traits: Ethical and Social Implications. Washington: Georgetown University Press, pp. 29–47.

[bibr24-1470594X18810439] Kagan S (2012) The Geometry of Desert. New York: Oxford University Press.

[bibr25-1470594X18810439] Kass LR (2003) Ageless bodies, happy souls: biotechnology and the pursuit of perfection. The New Atlantis 1: 9–28. 15584192

[bibr26-1470594X18810439] Levy N (2005) The good, the bad and the blameworthy. Journal of Ethics & Social Philosophy 1: 2–16.

[bibr27-1470594X18810439] Mehlman MJ (2004) Cognition-enhancing drugs. The Milbank Quarterly 82: 483–506. 15330974 10.1111/j.0887-378X.2004.00319.x PMC2690227

[bibr28-1470594X18810439] Milne H (1986) Desert, effort and equality. Journal of Applied Philosophy 3: 235–243.

[bibr29-1470594X18810439] Parens E (2005) Authenticity and ambivalence: toward understanding the enhancement debate. Hastings Center Report 35: 34–41. 16092400

[bibr30-1470594X18810439] Perry S (1992) The moral foundations of tort law. Iowa Law Review 77: 449–514.

[bibr31-1470594X18810439] President’s Council on Bioethics (2003) Beyond Therapy: Biotechnology and the Pursuit of Happiness. Washington: President’s Council on Bioethics.

[bibr32-1470594X18810439] Rachels J (1978) What people deserve In: Arthur J Shaw WH (eds), Justice and Economic Distribution. Prentice-Hall: Englewood Cliffs, pp. 150–163.

[bibr33-1470594X18810439] Roache R (2008) Enhancement and cheating. Expositions 2: 153–156.

[bibr34-1470594X18810439] Rose S (2006) The Future of the Brain: The Promise and Perils of Tomorrow’s Neuroscience. New York, Oxford: Oxford University Press.

[bibr35-1470594X18810439] Sadurski W (1985) Giving Desert Its Due: Social Justice and Legal Theory. Dordrecht: Springer Science & Business Media.

[bibr36-1470594X18810439] Sandel M (2007) The Case Against Perfection: Ethics in the Age of Genetic Engineering. Cambridge: Harvard University Press.

[bibr37-1470594X18810439] Santoni de Sio F Faber NS Savulescu J (2016) Why less praise for enhanced performance? Moving beyond responsibility-shifting, authenticity, and cheating to a nature of activities approach In: Jotterand F Dubljević V (eds), Cognitive Enhancement: Ethical and Policy Implications in International Perspectives. Oxford: Oxford University Press, pp. 27–38.

[bibr38-1470594X18810439] Santoni de Sio F Robichaud P Vincent NA (2014) Who should enhance? Conceptual and normative dimensions of cognitive enhancement. Humana Mente Journal of Philosophical Studies 26: 179–197.

[bibr39-1470594X18810439] Savulescu J (2006) Justice, fairness, and enhancement. Annals of the New York Academy of Sciences 1093: 21–38. 10.1196/annals.1382.021 17312266

[bibr40-1470594X18810439] Schermer M (2008a) Enhancements, easy shortcuts, and the richness of human activities. Bioethics 22: 355–363. 18445089 10.1111/j.1467-8519.2008.00657.x

[bibr41-1470594X18810439] Schermer M (2008b) On the argument that enhancement is “cheating.” Journal of Medical Ethics 34: 85–88. 18234944 10.1136/jme.2006.019646

[bibr42-1470594X18810439] Smilansky S (1996) Responsibility and desert: defending the connection. Mind 105: 157–163.

[bibr43-1470594X18810439] Sorensen K (2010) Effort and moral worth. Ethical Theory and Moral Practice 13: 89–109. 10.1007/s10677-013-9441-4 PMC4074890 24991189

[bibr44-1470594X18810439] Tobey DL (2003) What’s really wrong with genetic enhancement: a second look at our posthuman future. Yale Journal of Law and Technology 6: 54–160.

[bibr45-1470594X18810439] Watson G (1996) Two faces of responsibility. Philosophical Topics 24: 227–248.

[bibr46-1470594X18810439] Whitehouse PJ Juengst E Mehlman M (1997) Enhancing cognition in the intellectually intact. Hastings Center Report 27: 14–22. 9219019

